# Initiators counteract Polycomb repression and stimulate long-range contacts between enhancers and the *Abdominal-B* promoter in *Drosophila*

**DOI:** 10.1098/rsob.250192

**Published:** 2025-11-26

**Authors:** Olga Kyrchanova, Ksenia Kudryashova, Vasilisa Dubrovskaya, Airat Ibragimov, Paul Schedl, Pavel Georgiev

**Affiliations:** ^1^ Department of the Control of Genetic Processes, Institute of Gene Biology Russian Academy of Sciences, 34/5 Vavilov St., Moscow 119334, Russia; ^2^ Princeton University, Princeton, NJ, USA

**Keywords:** chromatin boundary, enhancer–promoter interaction, bithorax, Polycomb, tethering element, chromatin insulator

## Abstract

The specification of abdominal segments A5 to A9 depends on the expression of *Abdominal-B* (*Abd-B*), which is regulated by four *infraabdominal* domains: *iab-5* through *iab-8,9*. Each *iab* domain contains an initiation element that determines its active state, along with enhancers responsible for tissue-specific activation of *Abd-B*. These *iab* domains function autonomously due to their flanking boundaries, *Fab-6*, *Fab-7* and *Fab-8*, which both block crosstalk between adjacent *iab* domains (insulator function) and facilitate long-range interactions with the *Abd-B* promoter (bypass function). In inactive *iab* domains, enhancers are repressed by Polycomb group (PcG) proteins. Activation of the *iab* domains is driven by initiators, which are stimulated in a segment-specific manner by products of the gap and pair-rule genes during early embryogenesis. By creating truncations of the *Fab-6* boundary, we identified that the bypass module is adjacent to CTCF binding sites and overlaps with sequences responsible for recruiting PcG proteins. Using genome editing, we demonstrated that the *iab-5* and *iab-6* initiators enhance the activity of the *Fab-6* bypass module. Therefore, initiators represent a novel class of regulatory elements that control long-distance interactions between *iab* enhancers and *Abd-B* promoters.

**Figure:**
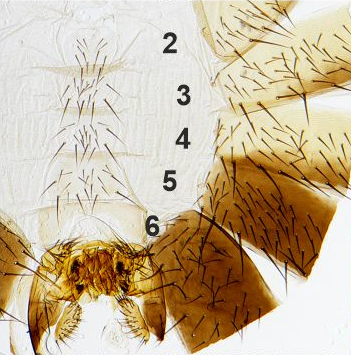

## 1. Introduction

In higher eukaryotes, the activation of promoters for many genes that primarily regulate the development of multicellular organisms is often controlled by numerous tissue-specific enhancers acting at particular developmental stages [1–3]. Enhancers are DNA regions, typically 100–1000 base pairs in length, containing binding sites for transcription factors that help stimulate transcription at promoters [4,5]. Enhancers recruit several key complexes, including a highly conserved mediator complex [6–8], and can activate transcription from promoters at large distances that may exceed 100 kb [1,2,8,9]. It is currently believed that promoters must be situated within a specific zone near enhancers to be activated, where enhancer-associated transcription factors and complexes form condensates that stimulate nearby promoters [2,10–13].

Recently, several classes of regulatory elements have been identified that form specific chromatin loops through long-distance interactions, bringing enhancers and promoters into the same active zone for functional communication [9,13]. In mammals, most studied long-range interactions are thought to be dependent on loops formed by cohesin complexes anchored at DNA-binding sites of CTCF and other architectural proteins [14,15]. In *Drosophila*, the interactions between insulators [16,17], Polycomb group (PcG) regulatory elements [18–22] and tethering elements [11,23,24] have been shown to regulate enhancer–promoter communication. Many chromatin loops between regulatory elements are stable and established prior to gene activation [25–27]. In other cases, enhancers come into proximity with gene promoters only in the specific cell types or developmental stages where the gene is expressed [27–31]. However, it remains unknown how the regulation of long-distance interactions occurs.

Among the most promising models for studying the regulation of long-distance interactions are clusters of homeotic (*Hox*) genes in mammals and *Drosophila* [11,32,33]. For instance, the distant regulatory elements that control expression of the mouse *HoxD13–HoxD10* gene cluster are separated from their target genes by multiple binding sites for the CTCF protein [34]. Long-range interactions between cohesin/CTCF sites have been shown to be modulated during the sequential activation of mouse *Hox* genes [35]. The *Drosophila* bithorax complex (BX-C), which includes three *Hox* genes *Ultrabithorax*, *abdominal-A* and *Abdominal-B (Abd-B)*, also serves as a convenient model system, as targeted genome editing is considerably easier in *Drosophila* than in mammals [36–41].

**Figure 1.**
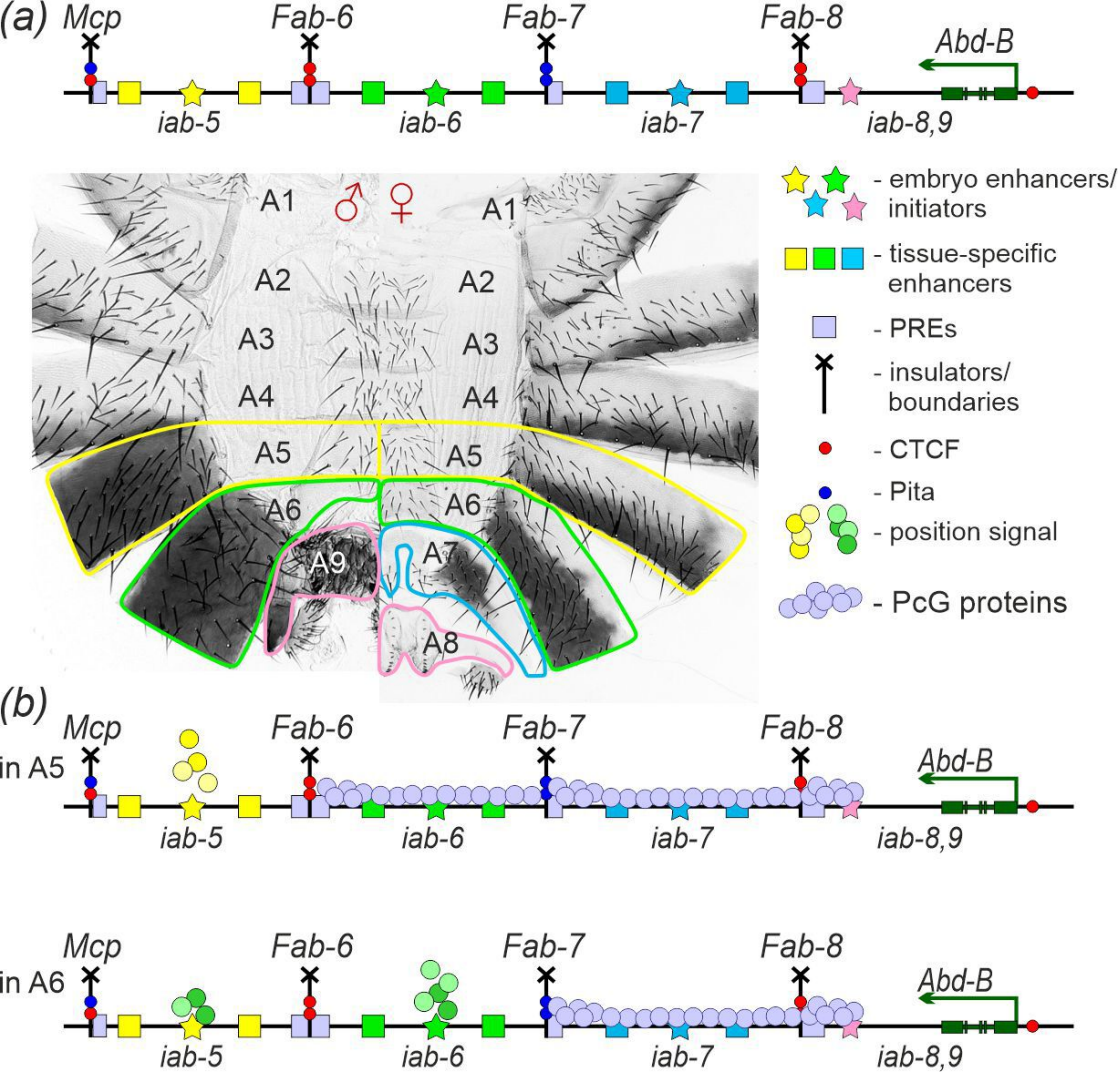
‘Open for business’ model of *Abd-B* gene expression regulation. (*a*) Diagram of the *Abd-B* regulatory region (not in scale). It contains four *cis*-regulatory domains that control *Abd-B* gene expression (the promoter direction is indicated by the green arrow). Each *iab* domain includes an initiator (shown as a star), which in transgene assays functions as a parasegmentally limited embryonic enhancer, tissue-specific enhancers (represented by coloured squares) and PREs (pale lilac rectangles). The *Mcp, Fab-6, Fab-7* and *Fab-8* boundaries flank the *iab* domains (depicted as vertical black lines with a cross on top). The *iab-5* domain (highlighted in yellow) specifies the abdominal segment A5, which is correspondingly marked on the adult male and female cuticles. The *iab-6* domain (marked in green) regulates *Abd-B* expression in the A6 segment. The *iab-7* domain (marked in blue) controls *Abd-B* expression in the A7 segment, which is absent in males and reduced in size in females. Finally, the *iab-8,9* domain (coloured pink) directs the development of genitalia: segment A8 in females and A9 in males. (*b*) Schematic of the ‘open for business’ model. The diagrams depict *Abd-B* expression in segments A5 (PS10) and A6 (PS11).

The most thoroughly studied regulatory region is that of *Abd-B*, which comprises the *iab-5*, *iab-6, iab-7* and *iab-8,9* domains. These domains control *Abd-B* expression in the A5 (PS10), A6 (PS11), A7 (PS12), A8 (PS13) and A9 (PS14) abdominal segments in adults (parasegments in the embryo; figure 1*a*) [11]. Each domain is arranged in the same order as the segment it is responsible for differentiating and provides the gradual increase in *Abd-B* expression, from the lowest in A5(PS10) to the strongest in A7(PS12)–A9(PS14), which determines the structural features of each segment. The *iab* domains contain early embryonic and tissue-specific enhancers, most of which have not yet been mapped and are isolated from each other by boundaries: *Mcp, Fab-6, Fab-7* and *Fab-8*, consisting of motifs for architectural proteins including well-described dCTCF and Pita [42–44]. These boundary elements have insulator activity and are essential for maintaining the functional autonomy of the *iab* domains [45–52]. Studies have shown that artificial sequences containing 4–5 binding sites for dCTCF or Pita can form insulators that effectively block crosstalk between enhancers in adjacent *iab* domains [43,44,53]. All four of the *Abd-B* boundaries are located near Polycomb response elements (PREs) [52,54,55], which recruit conserved among higher eukaryotes transcription repressors, PcG proteins [56–61].

According to the ‘open for business’ model (figure 1*b*), individual regulatory domains are repressed by PcG proteins and become activated (opened) only in the appropriate embryonic parasegments [38,62,63]. Inactivation of one of the PcG proteins leads to the derepression of bithorax genes across all body segments, highlighting the crucial role of PcG silencing in maintaining the inactive (*off*) state of the regulatory domains [54,64–67]. This model also explains why transgenes inserted into a regulatory domain are expressed only in segments where that domain is active [68,69]. In the open for business model, the activity state of the BX-C regulatory domains is set in early embryos by parasegment-specific initiation elements [70]. These elements are thought to ‘read’ a parasegmental address, determined by specific concentrations of maternal, gap and pair-rule gene products, and relay this information to the other regulatory elements within the domain [62,71]. Consistent with this model, elements that function as early embryonic enhancers that respond to the parasegmental addresses of the *iab-5–iab-8* regulatory domains were identified in transgene assays [71–78]. Further support for this model came from the experiments, demonstrating that the deletion of the *iab-6* embryonic enhancer (initiator) led to the transformation of the A6 segment into a copy of A5 [79]. When the *iab-6* initiator was replaced by the *iab-5* embryonic enhancer, the *iab-6* domain was turned on in A5, converting this segment into a copy of A6 [79]. At later embryonic stages, the maintenance of the *on* state depends on proteins from the Trithorax (Trx) group [38,56]. According to this model, the primary role of boundaries is to prevent crosstalk between enhancers and PREs located in adjacent *iab* domains.

**Figure 2.**
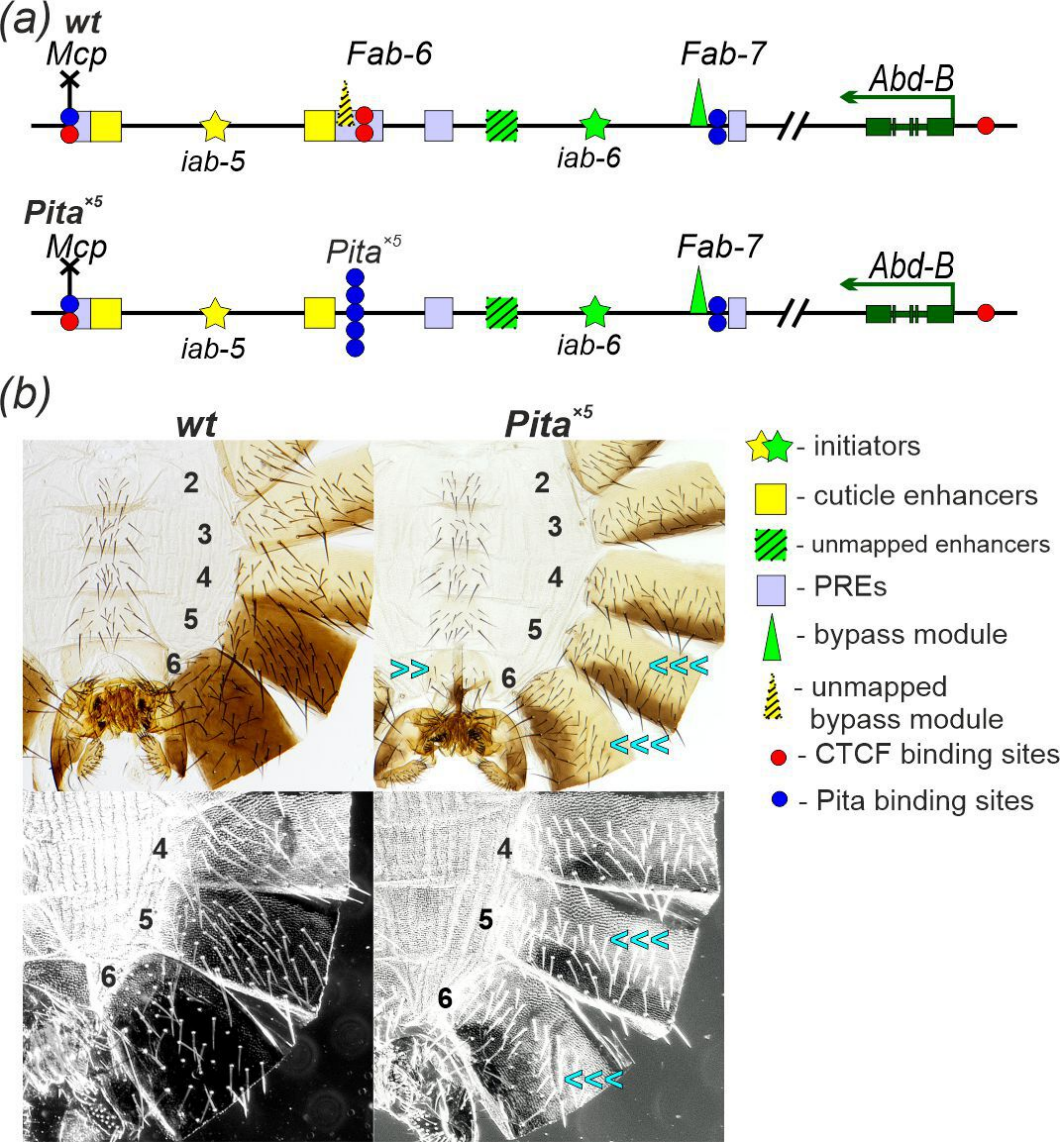
The model system used to test the functional role of the *iab-5* and *iab-6* initiators. (*a*) Schematic of the *iab-5* and *iab-6* regulatory domains in *wild-type* (*wt*) and *Pita^×5^* flies. The mapped tissue-specific (cuticle) enhancers—responsible for pigmentation of the tergites, suppression of trichome hairs on the tergites and bristles on the sternites and reduction of the sternite—are marked by yellow squares. Tissue-specific enhancers within *iab-6*, which are responsible for suppression of trichome hairs on the tergites and bristles on the sternites, as well as sternite reduction, have not yet been mapped and are indicated by a hatched green square. Boundary bypass modules are represented by triangles: *Fab-7* (green) and *Fab-6* (hatched yellow). In *Pita^×5^* flies, the *Fab-6* boundary is replaced with an artificial *Pita^×5^* insulator, depicted as a column of five blue circles. Other designations are as in figure 1. (*b*) Comparison of the morphology of the abdominal A5 and A6 segments (numbered) in *wt* and *Pita^×5^* males. Trichomes on the A5 and A6 tergites are shown below in the dark field. In *wt* males, tergites A5 and A6 are pigmented. The A5 sternite has a quadrilateral shape and is covered with bristles, whereas the A6 sternite has a distinct shape and lacks bristles. The A6 tergite displays trichomes along the anterior and ventral edges, whereas the entire A5 tergite is generally covered in trichomes, except for a small internal patch that lacks them. In *Pita^×5^* males, both A5 and A6 tergites show a mostly complete loss of pigmentation. On the A5 tergite, trichomes are densely packed, similar to those on the A4 tergite. Trichomes also cover the entire A6 tergite. Triple, double and single blue arrows indicate nearly complete, moderate and weak loss of specific individual features of segments A5 and A6, respectively, which correlates with a decrease in *Abd-B* expression.

Recently, we found that the *Fab-7* and *Fab-8* boundaries have two activities, a blocking or insulator function and an ability to facilitate specific long-distance interactions between enhancers in the *iab* regulatory domains and *Abd-B* promoters [80–82]. Bypass (also called tethering) elements have been mapped within both boundaries (figure 2*a*). Micro-C studies in embryos revealed physical interactions between the *Fab-7* and *Fab-8* boundaries and the *Abd-B* promoter region [23], suggesting that their bypass modules interact with corresponding elements in the *Abd-B* promoter region. Additionally, it was shown that the order of the boundary bypass and blocking modules in the *Fab-7* and *Fab-8* boundaries relative to the *iab* initiator is critical for bypass activity: the bypass module cannot be separated from the initiator by the insulator module [83]. These findings suggest a model in which initiators directly activate the bypass modules to establish long-distance interactions with the *Abd-B* promoter region.

In this study, we combined CRISPR/Cas9 and *φC31*-mediated recombination approaches to investigate the regulatory function of the *Fab-6* boundary and to clarify the role of initiators in orchestrating the ‘open for business’ model of *iab* domains. We found that the *Fab-6* bypass module overlaps with PRE sequences in *iab-5*, and its activity appears to be regulated by this PcG silencer. This overlap with the silencer enables the nearby enhancer to be repressed in segments where the regulatory domain should be inactive. The *iab-5* and *iab-6* initiators serve two key functions during *iab* domain activation: they counteract PcG silencing and stimulate the bypass modules to organize long-distance contacts between the activated *iab* domains and the *Abd-B* promoter.

## 2. Results

### 2.1. Model system for studying the role of the initiator in regulating the *Fab-6* boundary

To investigate the functional roles of initiators and boundaries, we focused on the male abdominal segments A5 and A6. These two segments have distinct morphological features (figures 1 and 2) and alterations in their morphology reflect changes in the functioning of their corresponding regulatory domains, *iab-5* and *iab-6*.

The approximately 12 kb *iab-5* domain (figure 2*a*) is flanked by the *Mcp* and *Fab-6* boundaries, which confer its autonomy. The neighbouring distally located *iab-6* domain of approximately 15 kb is flanked by the *Fab-6* and *Fab-7* boundaries (figure 2*a*). The *Mcp* boundary separates the *abd-A* and *Abd-B* regulatory regions and lacks a bypass module [82]. The *Mcp* consists of an insulator and a nearby 135 bp PRE [84] located on the *iab-5* side. The *Fab-7* boundary includes the bypass element, insulator and PRE [55,80]. PRE is involved in insulator function of the *Fab-7* boundary [85]. The *Fab-6* boundary separates the *iab-5* and *iab-6* domains and exhibits an unusual structural organization. While the core of the *Fab-6* boundary was mapped to a 529 bp region, full insulator activity requires an additional PRE located more than 4 kb inside the *iab-6* domain [52,86]. The core part of the *Fab-6* boundary contains two CTCF binding sites [42,87]. Notably, this region shows repressor activity regulated by PcG proteins [52]. Although the presence of a bypass module has been predicted within the *Fab-6* boundary, it has not yet been mapped. The 1 kb *iab-5* initiator (*i5*) is positioned approximately 2.6 kb from the *Fab-6* boundary [54]. Two tissue-specific enhancers located near the *Mcp* and *Fab-6* boundaries drive *Abd-B* activation in the cuticle of the A5 and A6 segments [88]. The *iab-6* domain contains a tissue-specific enhancer, the exact location of which has not been determined [79]. The *iab-6* initiator (*i6*) is localized to a 900 bp region approximately 5 kb from the *Fab-7* boundary [79].

*Abd-B* is not expressed in segments A2 to A4, and their development is controlled by *abd-A*. In *wild-type* (*wt*) males, the surface of the tergites in these segments is densely covered with trichomes, and pigmentation is limited to a stripe along the distal edges of each segment [89] (figure 2*b*). Activation of *Abd-B* in A5 and A6 alters their morphology in males. Both tergites are fully pigmented. The low level of *Abd-B* expression in the male A5 segment weakly suppresses trichome development, and the trichome density is noticeably less than in A4 and more anterior tergites. On the other hand, the morphology of the A5 sternite resembles the A4 sternite; it has a quadrilateral shape and is covered with bristles. In A6, *Abd-B* expression is driven by the same *iab-5* enhancers along with the predicted *iab-6* tissue-specific enhancer. The higher level of *Abd-B* expression in A6 leads to an almost complete suppression of trichomes across most of the tergite surface, and the trichomes are restricted to the anterior and ventral edges. It also alters the morphology of sternite, which has a crescent shape and is devoid of bristles (figure 2*b*).

Replacement of the *Fab-6* boundary with an insulator consisting of five Pita binding sites (*Pita^×5^*) blocks the *iab-5* domain from regulating *Abd-B*, causing the A5 segment to transform into a copy of A4 (A5→A4): the tergite becomes unpigmented and densely covered with trichomes (figure 2*b*). Because the *iab-5* enhancers also contribute to *Abd-B* expression in A6, *Pita^×5^* males exhibit a pronounced phenotypic transformation of the A6 segment into A4 as well: trichomes are densely distributed across the unpigmented tergite, and the sternite is thicker and has several bristles. However, the pigment spots lacking trichomes frequently appear on the A6 tergite, suggesting that the *iab-5* enhancers retain a weak ability to activate *Abd-B*. Thus, the mapped *iab-5* enhancers play a substantial role in *Abd-B* expression within the cuticular structures of the male A6 segment.

### 2.2. Mapping the *Fab-6* bypass module

**Figure 3.**
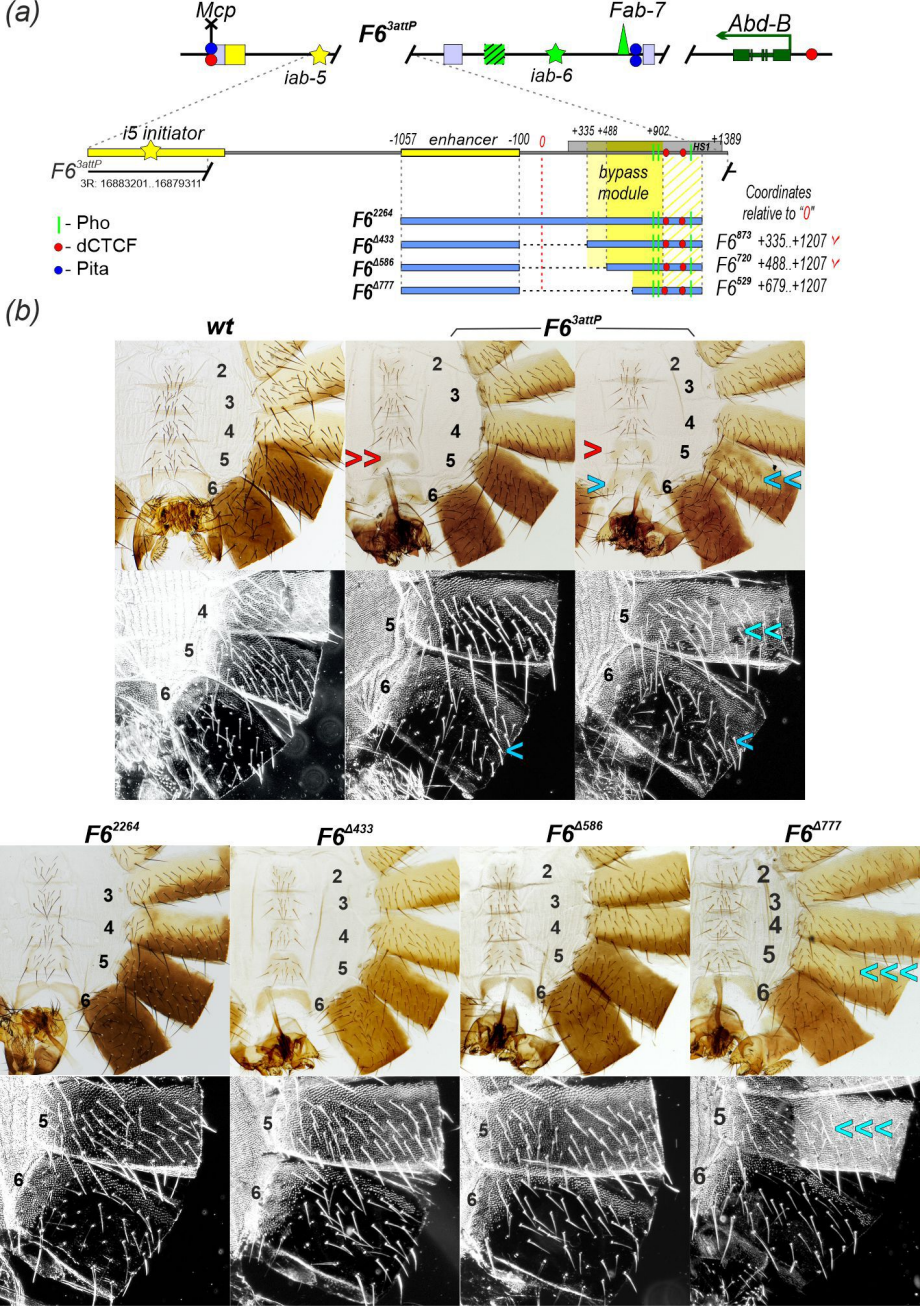
Mapping the bypass module at the *Fab-6* boundary in the *F6^3attP^* platform. (*a*) Schematic of *F6^3attP^* substitutions. In the *F6^3attP^* platform, the DNase I hypersensitive region (HS1, shown as thick grey line) of the *Fab-6* boundary and adjacent cuticle enhancer (shown as thick yellow line) is deleted and replaced with an *attP* site. The endpoints of the *F6^3attP^* are indicated by black lines, and the coordinates are according to the FlyBase, Dmel. r6.62. The *Fab-6* derivatives used for replacement are shown as thick blue lines, with fragment coordinates given relative to the proximal edge (0 point) of the *F6^1attP^* deletion [86]. The area shaded with pale-yellow lines defines the approximate boundaries of the complete bypass module, while the richer yellow defines the core of the bypass module. Fragments that have bypass activity are marked with a red check mark. dCTCF binding sites are indicated by red circles, Pho sites—by vertical green lines. Fragments that have bypass activity are indicated with a red check mark. Other designations are as in figure 2. (*b*) Morphology of male abdominal segments (numbered) in *F6^3attP^* replacements. Bright- and dark-field images of cuticles prepared from 2- to 3-day-old males of the obtained lines. The photo of the *wt* is the same as in figure 2*b*. *F6^3attP^* displays a variable phenotype resembling both previously described *Fab-6* deletions. The integration of F*6^Δ433^* and *F6^Δ586^* restored the morphology of the male abdominal segments to *wt*. In contrast, in *F6^Δ777^*, the morphology of A5 is identical to A4 (indicated by triple blue arrows), whereas the morphology of A6 resembles *wt*. A detailed description of the morphological features of the A5 and A6 segments is given in the caption to figure 2*b*.

Since bypass modules of the *Fab-7* and *Fab-8* boundaries flank the *iab-6* and *iab-7* domains, respectively [80,81], we expected that the *Fab-6* bypass module would be located on the *iab-5* side of the *Fab-6* boundary. To study the functional activity of different truncated versions of *Fab-6*, we created a new *F6^3attP^* platform in which a 3892 bp region including the *Fab-6* boundary [86] and an adjacent (*iab-5*) cuticle enhancer [88] was deleted and replaced with *attP* site (figure 3*a*). Insertion of a 2664 bp fragment (*F6^2664^*) [86] that includes the cuticle enhancer [88] and the *Fab-6* boundary element completely restored the *wt* phenotype. Thus, the 1445 bp deletion of the sequences between the *i5* initiator and the enhancer is not essential for the *iab-5* functions. We then made a series of internal deletions extending from the distal end of the cuticle enhancer (figure 3*b*). The 433 bp (*F6^Δ433^*) and 586 bp (*F6^Δ586^*) deletions, which retain 873 bp (*F6^873^*) and 720 bp (*F6^720^*) of *Fab-6*, respectively, do not affect the proper development of the A5 segment. In contrast, a 777 bp deletion (*F6^Δ777^*) leads to an almost complete transformation of the A5 segment into a copy of A4. Interestingly, *F6^Δ777^* coincides with the core region of the boundary (*F6^529^*), which is capable of maintaining normal *iab-5* domain activity. This suggests that *F6^Δ777^* removes only a portion of the *Fab-6* bypass module.

To further confirm the identity of the *Fab-6* sequences that are responsible for enabling the *iab-5* domain to activate *Abd-B* expression, we used the experimental approach that had previously been successful in mapping the bypass modules of the *Fab-7* and *Fab-8* boundaries [80,81]. The approximately 200 bp bypass modules in *Fab-7* and *Fab-8* were mapped by combining different parts of the boundaries with the *Pita^×5^* insulator. We found that artificial boundaries consisting of either the *Fab-7* or *Fab-8* bypass modules and *Pita^×5^* fully perform the dual functions of *Fab-7*, blocking crosstalk between adjacent domains and ensuring proper activation of *Abd-B* by *iab-6* enhancers.

**Figure 4.**
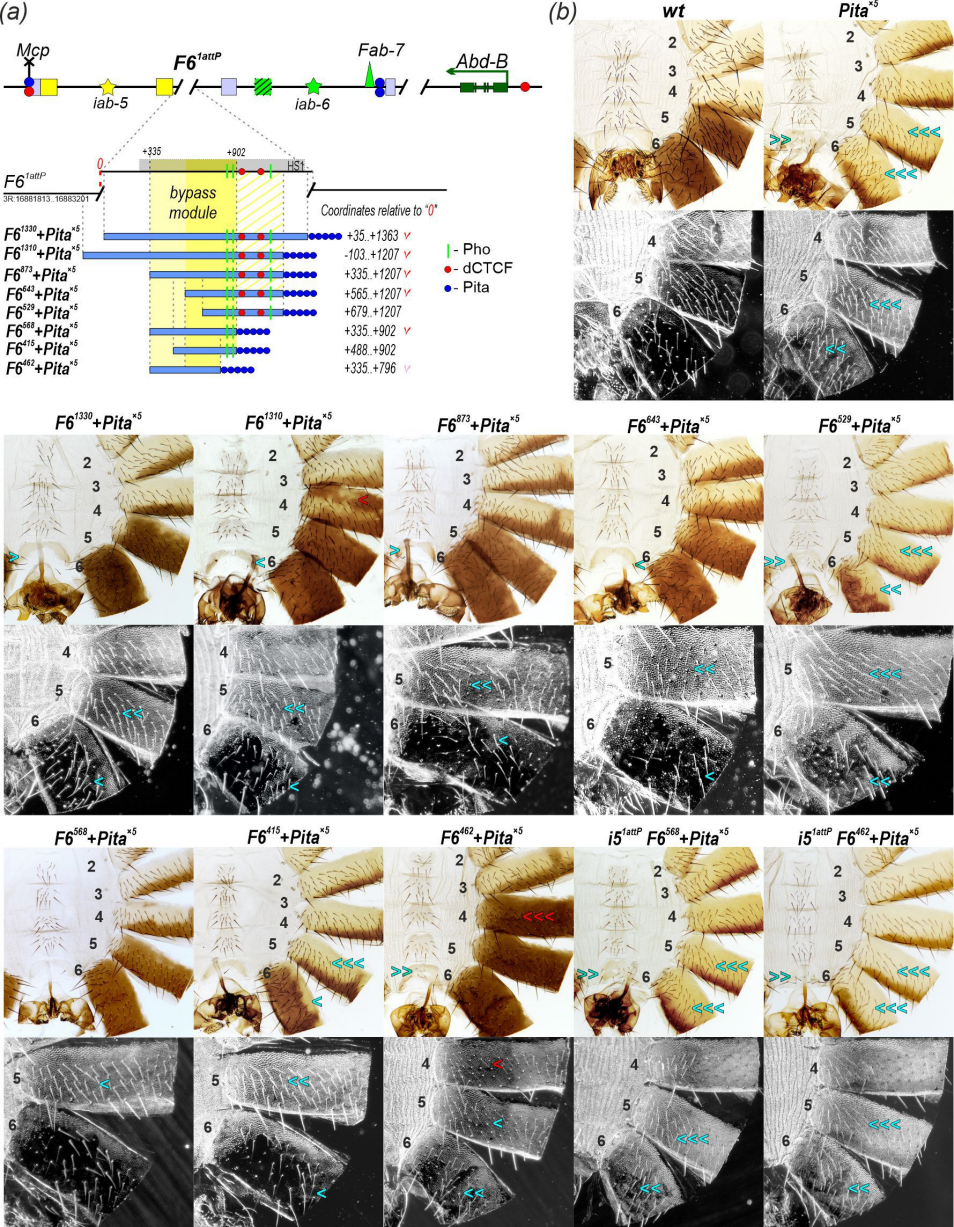
Mapping of the bypass module in the *Fab-6* boundary. (*a*) Schematic of the *iab-5* and *iab-6* domains along with *Fab-6* substitutions. The *F6^1attP^* platform used for replacements is indicated by dashed black lines. The coordinates of the *F6^1attP^* endpoints correspond to FlyBase, Dmel. r6.62. *Fab-6* truncated derivatives are shown as thick blue lines, with fragment coordinates given relative to the proximal edge of the deletion. *Pita^×5^* is depicted as five blue circles. The thick grey line marks the DNase I hypersensitive region (HS1). The pale-yellow area indicates the location of the bypass module. The area hatched with pale-yellow lines indicates the area that supports the truncated bypass module (a richer yellow). Fragments that have bypass activity are marked with a red check mark. A pink check mark designates unstable bypass activity. Pho sites are represented by vertical green lines. (*b*) Bright- and dark-field images of cuticles prepared from 2- to 3-day-old males of the generated lines. A detailed description of the morphological features of the A5 and A6 segments is given in the caption to figure 2. The morphology of abdominal segments A5 and A6 (numbered) in lines with truncated *Fab-6* derivatives in combination with the *Pita^×5^* insulator has some deviations, which are indicated by arrows. Triple, double and single blue arrows indicate nearly complete, moderate and weak loss of specific features in segments A5 and A6, correlating with a reduction in *Abd-B* expression. Triple, double and single red arrows denote strong, moderate and weak ectopic *Abd-B* expression, respectively. The photo of the *wt* is the same as in figure 2*b*. Other designations are as in figure 2.

For this analysis, we used a previously generated *F6^1attP^* replacement platform [86], in which a 1389 bp region of *Fab-6* was substituted by an *attP* site (figure 4*a*). As noted above, insertion of the Pita^×5^ insulator into *F6^1attP^* (*Pita^×5^*) results in a complete inactivation of *Abd-B* expression in A5 and partial inactivation in A6 (figure 4*b* and electronic supplementary material, figure S1). We first tested whether the complete *Fab-6* boundary could mediate the functional activity of *iab-5* in the presence of the *Pita^×5^* insulator. For this, we tested two *Fab-6* boundary fragments, 1310 and 1330 bp (*F6^1310^* and *F6^1330^*), which cover nearly the entire deleted region. *F6^1310^* overlaps by 103 bp with the sequence near the proximal breakpoint of the *F6^1attP^* deletion, while *F6^1330^* is 138 bp shorter at the proximal edge and 156 bp longer at the distal edge compared to *F6^1310^* (figure 4*a*). Both fragments contain two CTCF binding sites and three potential binding sites for Pleiohomeotic (Pho), a known recruiter of PcG proteins [90–92].

The combinations *F6^1310^ + Pita^×5^* and *F6^1330^ + Pita^×5^* substantially, though not completely, restore *iab-5* activity in segments A5 and A6 (figure 4*b* and electronic supplementary material, figure S1). In these males, trichome hairs on the A5 tergite are more densely packed than in *wt* males, while ectopic trichomes appear on the A6 tergite along with a few scattered bristles on the A6 sternite. Interestingly, males carrying *F6^1310^+Pita^×5^* often exhibit mosaic pigmentation on the A4 tergite, indicating ectopic *Abd-B* activation (figure 4*b* and electronic supplementary material, figure S1). This ectopic pigmentation is observed much less frequently in *F6^1330^ + Pita^×5^* males. Taken together, these findings suggest that both *F6^1310^* and *F6^1330^* fragments contain a bypass module capable of partially overcoming the strong blocking activity of the *Pita^×5^* insulator.

We then generated three artificial boundaries with proximal truncations of the *F6^1310^* fragment: *F6^873^ + Pita^×5^*, *F6^643^ × Pita^×5^* and *F6^529^ × Pita^×5^* (figure 4*a*). Interestingly, the cuticle phenotypes of A5 and A6 in *F6^873^ + Pita^×5^* and *F6^643^ + Pita^×5^* males more closely resemble the *wt* than those in *F6^1310^ + Pita^×5^* males (figure 4*b* and electronic supplementary material, figure S1). Notably, there is a considerable reduction in ectopic trichrome hairs on the A6 tergite, and no mosaic pigmentation is observed on the A4 tergite. These results suggest that the bypass module is retained in both truncated replacements. Because neither *F6^873^ + Pita^×5^* nor *F6^643^ + Pita^×5^* induces pigmentation of the A4 tergite or extensive ectopic trichomes in the A6 tergite, it is reasonable to assume that there is a regulatory element in sequences at the proximal end of *F6^1310^* that counteracts the silencer in *Fab-6*. Again, the smaller *F6^529^ + Pita^×5^* replacement appears to lack bypass activity, as the phenotype of adult *F6^529^ + Pita^×5^* males resembles that of *Pita^×5^* alone. Thus, *F6^529^ + Pita^×5^* loses the ability to maintain *iab-5* domain activity, as shown above for *F6^Δ777^*. These results are consistent with the suggestion that bypass module activity is partially impaired in *F6^529^*, and its activity is not sufficient to overcome the blocking function of the *Pita^×5^* insulator.

To localize the distal end of the bypass module, we generated *F6^568^ + Pita^×5^* and *F6^415^ + Pita^×5^* boundary variants that lack the two *Fab-6* CTCF binding sites but retain two Pho protein-binding sites (figure 4*a*). The proximal endpoint of *F6^568^* coincides with *F6^873^*, and the proximal endpoint of *F6^415^* coincides with *F6^720^*. The phenotype of the *F6^568^ + Pita^×5^* replacement was similar to that of *F6^873^ + Pita^×5^* and *F6^643^ + Pita^×5^*, closely resembling the *wt* (figure 4*b* and electronic supplementary material, figure S1). Surprisingly, the *F6^415^ + Pita^×5^* replacement lacks bypass activity in A5 but retains a clearly reduced bypass function in A6: A5 transforms into A4, whereas A6 remains closer to the *wt*. Given that the proximal endpoint of *F6^415^* extends beyond *F6^643^* but exhibits less bypass activity, this suggests that the deleted distal sequences, including the CTCF sites, may enhance bypass function.

Adjacent to the proximal CTCF site are two motifs for the Pho protein, suggesting that this region may be involved in the recruitment of PcG proteins. To test this possibility and map the bypass module from the distal side, we generated a truncation *F6^462^*, which removes the two *Fab-6* Pho binding sites but retains the same proximal endpoint as *F6^568^* (figure 4*a*). The *F6^462^ + Pita^×5^* replacement results in a pigmented A4 tergite, although it still has densely packed trichome hairs. Additionally, trichomes on tergites A5 and A6 are more densely distributed (figure 4*b*). These results suggest that the bypass activity in *F6^462^ + Pita^×5^* is partially compromised. On the other hand, the results confirm that the region containing the Pho sites is involved in the recruitment of PcG proteins that suppress the premature activation of the *iab-5* domain in A4. Taken together, these findings indicate that the bypass module is localized within a 568 bp region (+335 to +902; figure 4*a*) that partially overlaps with the PRE responsible for recruiting PcG proteins.

Next, we asked whether the ectopic pigmentation in A4 depended on the initiator *i5*. To test this, we deleted the *i5* initiator in the *F6^462^ + Pita^×5^* and *F6^568^ + Pita^×5^* lines (figure 4*b* and electronic supplementary material, figure S1). As a result, almost all *i5^1attP^ F6^568^ + Pita^×5^* and *i5^1attP^ F6^462^ + Pita^×5^* males showed complete loss of pigmentation in both the A5 and A6 segments, with A4 pigmentation also lost in *i5^1attP^ F6^462^ + Pita^×5^* males. Occasionally, small, pigmented spots appeared on A6 tergites in males from both lines (electronic supplementary material, figure S1). These findings demonstrate that the *i5* initiator is essential for *iab-5* enhancer activity, even when PcG-mediated function is inactivated. These results mean that the *i5* initiator is required not only to suppress PcG-mediated repression, but also to organize an active *iab-5* domain. This supports a model in which initiators activate bypass modules to establish long-range interactions with the *Abd-B* promoter region.

In 16 h embryos, *Abd-B* expression is activated by the *iab* domains in the embryonic central nervous system (CNS). Unmapped enhancers in the *iab-5* and *iab-6* domains drive weak *Abd-B* expression in PS10 and stronger expression in PS11 (electronic supplementary material, figure S2). Previously, we showed that the bypass modules of the *Fab-7* and *Fab-8* boundaries enable CNS enhancers to activate *Abd-B* across the *Pita^×5^* insulator [83]. However, *Abd-B* expression is absent in PS10 of *F6^1310^ + Pita^×5^* embryos, and the same is observed in *F6^568^ + Pita^×5^* and *F6^415^ + Pita^×5^* embryos. These results indicate that the *Pita^×5^* insulator blocks the CNS enhancers in embryos regardless of whether the *Fab-6* boundary or its bypass module is present.

### 2.3. Role of the initiators in activating tissue-specific enhancers of the *iab-5* domain

**Figure 5.**
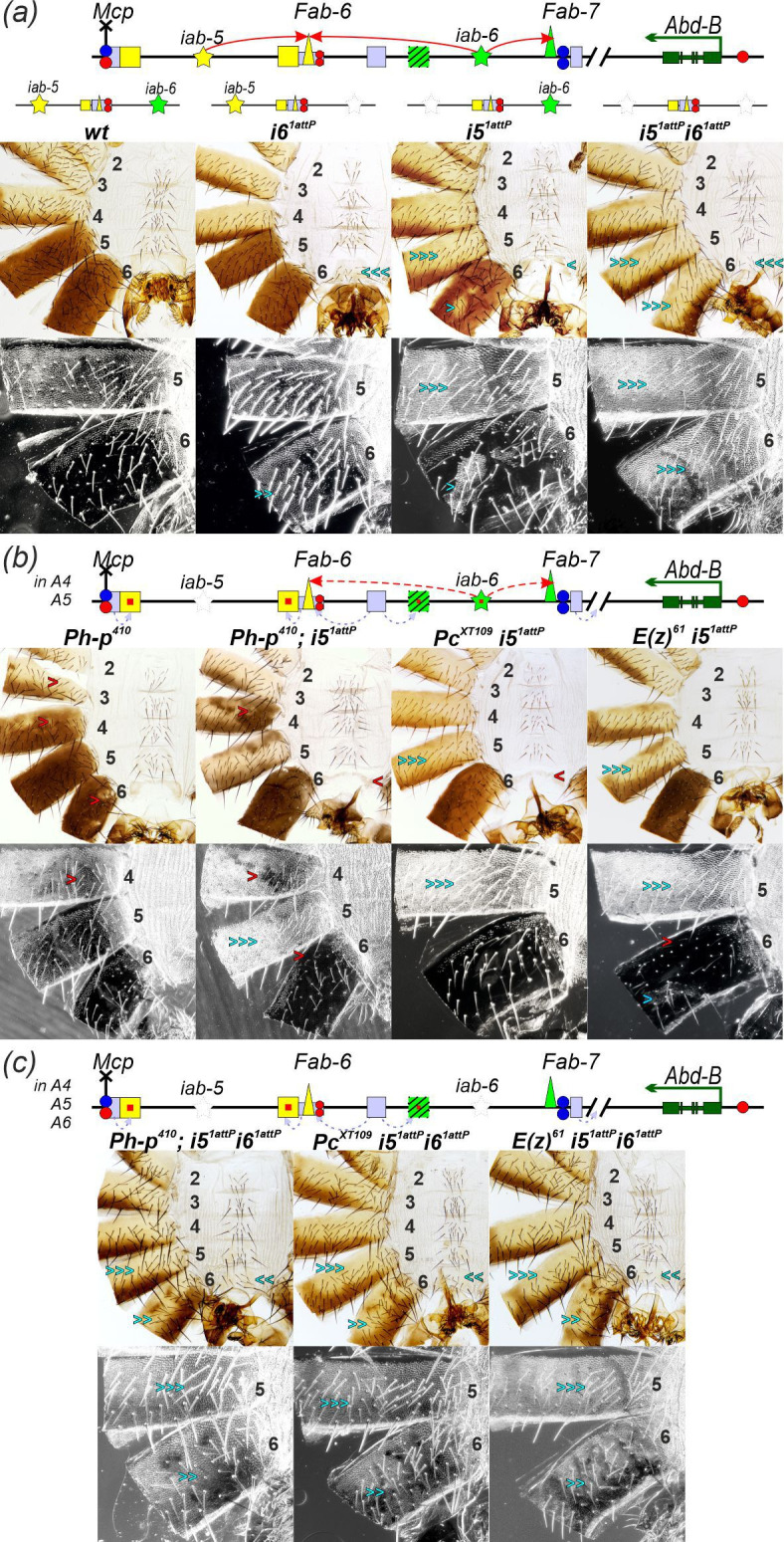
Functional role of the initiators in suppression of PcG-mediated silencing of the *iab-5* enhancers. (*a*) Schematic of the *iab-5* and *iab-6* domains and the deletions utilized in this experiment. Designations follow those in figures 2 and 3. A small red square indicates derepression of regulatory elements (enhancers or initiators) due to *ph-p^410^* mutation. The inability of PRE silencers to effectively repress enhancers is shown by the pale lilac dotted arrows. Red arc arrows indicate the activation of the bypass modules by the domain initiator. Dotted red arc arrows indicate the variable activation of the bypass modules, which is dependent on the level of initiator derepression. (*b*) Morphology of male abdominal segments in the *wt* and in males carrying the *i5^1attP^, i6^1attP^* or combined *i5^1attP^ i6^1attP^* initiator deletions. (*c*) Morphology of abdominal segments in *ph-p^410^/Y* males and in males with the *i5^1attP^* initiator deletion combined with mutations in PcG proteins: *ph^410^/Y; i5^1attP^/i5^1attP^, Pc^XT109^ i5^1attP^/i5^1attP^* and *E(z)^61^ i5^1attP^/i5^1attP^*. (*d*) Morphology of abdominal segments in males with deletions of both *i5^1attP^* and *i6^1attP^* initiators combined with PcG protein mutations: *ph^410^/Y; i5^1attP^ i6^1attP^/i5^1attP^ i6^1attP^, Pc^XT109^ i5^1attP^ i6^1attP^/i5^1attP^ i6^1attP^* and *E(z)^61^ i5^1attP^ i6^1attP^/i5^1attP^ i6^1attP^*. The photo of the *wt* is the same as in figure 2*b*.

It has been previously shown that the deletion of the *i6* initiator [79] results in a transformation of A6 into a copy of A5 (figure 5*a*). At the same time, the deletion of the *i5* initiator in *i5^1attP^* [88] leads to a complete transformation of the male A5 segment into a copy of A4 and a partial transformation of the A6 segment into A5 (figure 5*a*). These outcomes are explained by the role of tissue-specific *iab-5* enhancers in activating *Abd-B* in the A6 segment. Particularly notable is the pigmentation pattern of the A6 tergite because the enhancers controlling pigmentation reside exclusively within the *iab-5* domain. In *i5^1attP^* males, the A6 tergite exhibits mosaic pigmentation, often characterized by large unpigmented patches. These results suggest that the *iab-5* enhancers remain partially active in A6 despite the deletion of the *i5* initiator. The mosaic pigmentation likely reflects partial repression of the *iab-5* enhancers by PcG proteins that function in the absence of the initiator. These findings contradict the ‘open for business’ model, which proposes that without initiators, the domain is fully repressed by PcG proteins, but support the idea that initiators act to counteract Polycomb-mediated repression during activation.

To investigate the antagonism between PcG repression and *iab-5* enhancer activity in the *i5^1attP^* A6 segment, we introduced PcG mutations into the *i5^1attP^* deletion background: *ph-p^410^* (inactivation of the *proximal polyhomeotic* gene, *ph-p* [93]), *Pc^XT109^* (null mutation in the *Polycomb* gene [94]) and *E(z)^61^* (heat-sensitive loss-of-function point mutation (C603Y) in the *Enhancer of zeste* (*E(z*)) gene) [95]. Ph and Pc were shown to be required to maintain *Hox* gene silencing in embryo [96]. The *polyhomeotic* (*ph*) locus consists of two independent genes, *ph* proximal (*ph-p*) and *ph* distal (*ph-d*), which are closely related and appear to be functionally redundant [97]. The *ph* locus is on the X chromosome, and males carrying the *ph-p^410^* mutation are viable but exhibit partial gain-of-function transformations due to the loss of *ph-p* gene activity: A4 into A5 (resulting in pigmented A4 segments) and A6 into A7 (characterized by a reduced size of the A6 tergite; figure 5*b*). The *Pc^XT109^* and *E(z)^61^* are homozygous lethal, while heterozygous males show no obvious effects on abdominal development (data not shown).

As observed in *i5^1attP^*/*i5^1attP^*, the A5 abdominal segment in *i5^1attP^ Pc^XT109^/i51^1attP^* and *i5^1attP^ E(z)^61^/i51^1attP^* males is transformed into a copy of A4. However, heterozygosity for either *Pc^XT109^* or *E(z)^61^* suppresses the loss-of-function transformation of A6 towards A5 that is observed when *i5* initiator is deleted. Specifically, the A6 tergite was fully pigmented, trichomes appeared only along the anterior and ventral edges and the sternite was reduced to a thin strip without any bristles (figure 5*b*). These results show that PcG proteins mediate the partial repression of *iab-5* enhancers in the A6 segment of *i5^1attP^* males. Thus, when the activity of PcG proteins is partially disrupted by mutations, the *i5* initiator is critical for the activity of *iab-5* enhancers in A5, but not in the A6 segment. Surprisingly, in *ph-p^410^*/*Y; i5^1attP^* males, mosaic pigmentation is often observed on the tergite of segment A4, while it is only rarely observed on the A5 tergite (figure 5*b* and electronic supplementary material, figure S3). This result can be explained by a premature partial activation of the *i6* initiator, which is expected to be most active in odd parasegments [70].

Thus, when *iab-5* cuticle enhancers are derepressed by a reduction in PcG-dependent silencing, the *i6* initiator is somehow able to stimulate the activity of the bypass element to form long-range contacts between the *iab-5* cuticle enhancers and the *Abd-B* promoter. A model in which the *i6* initiator is able to stimulate the activity of *iab-5* enhancers independent of the *i5* initiator is supported by a comparison of the phenotypic effects of the *i5^1attP^* initiator deletion and the replacement of the *Fab-6* boundary by *Pita^×5^*. The phenotypic transformation of A6 towards an A4 identity is more complete in the *Pita^×5^* replacement (figures 2*b* and 4*b*) than in the *i5^1attP^* deletion (figure 5*a*).

To test the idea that the *iab-6* initiator facilitates *iab-5* enhancer activity in the A6 segment, we deleted the *i6* initiator in the *i5^1attP^* line (*i5^1attP^ + i6^1^* attP; figure 5*a*). The simultaneous deletion of both initiators causes the A5 and A6 male segments to transform into phenotypic copies of A4 (figure 5*a*). This demonstrates that the *i6* initiator is essential for the activity of the *iab-5* enhancers in the A6 segment. To further confirm this, we analysed the phenotype of *i5^1attP^i6^1attP^* males combined with PcG gene mutations. In contrast to *i5^1attP^*, the PcG mutations do not alter the phenotype of *i5^1attP^ i6^1attP^* males (figure 5*c* and electronic supplementary material, figure S3). Thus, it is the *i6* initiator that enables the *iab-5* enhancers to function in segments A6, A5 and A4 after removal of the *i5* initiator.

### 2.4. Is the activity of the *Fab-6* bypass module subject to regulation by the *iab-5* and *iab-6* initiators?

**Figure 6.**
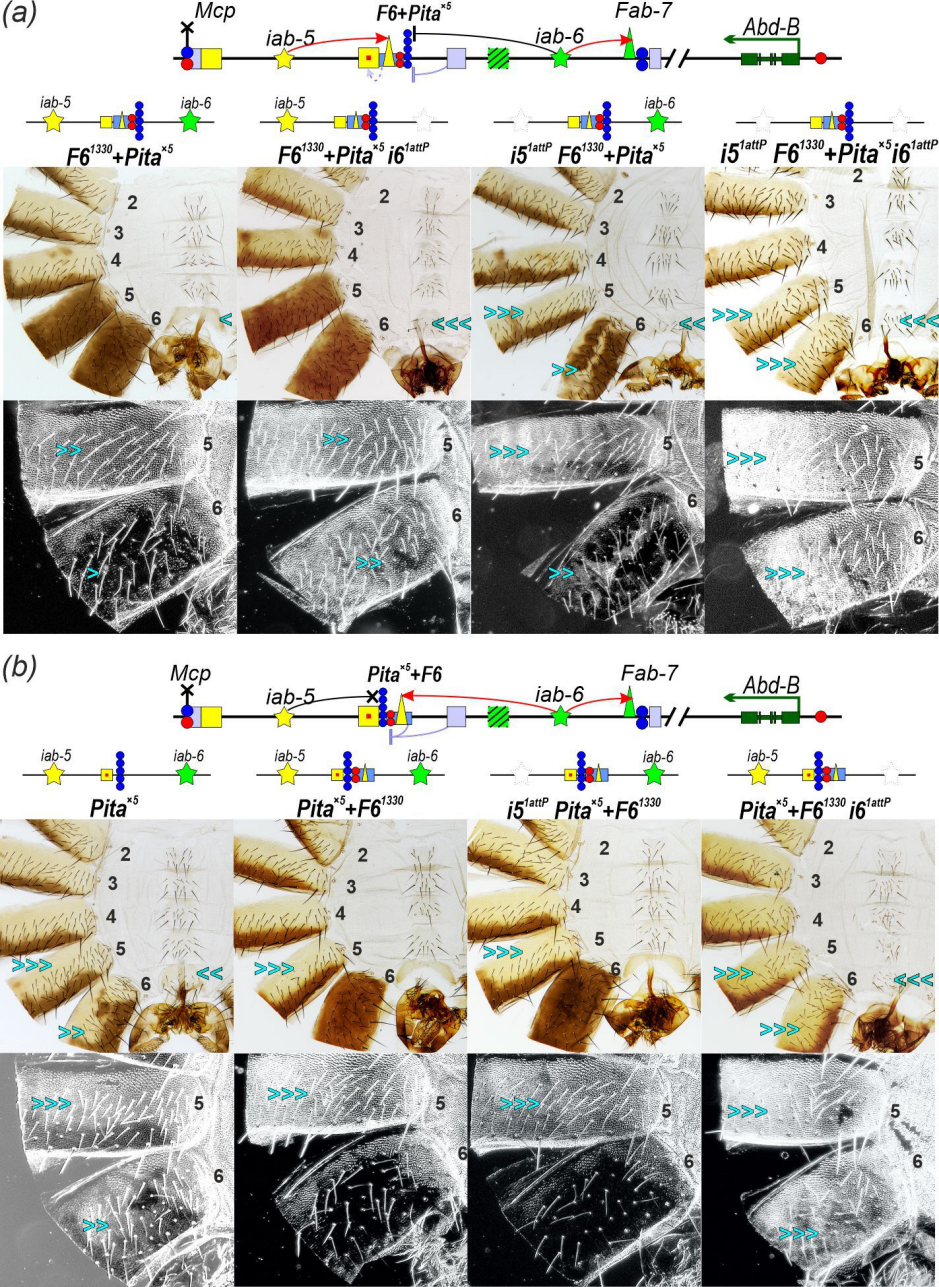
Functional role of the initiators in regulating the *iab-5* and *iab-6* domains. (a) Schematic of the *iab-5* and *iab-6* domains and the deletions used in the experiments. Red arc arrows indicate the activation of the bypass modules by the domain initiator. The black arc with a block at the end indicates that the activation signal from the *iab-6* initiator to the *Fab-6* bypass module is blocked by the *Pita*^*×5*^ insulator. A small red square indicates derepression of enhancers due to blocking *Fab-6* PRE by *Pita*^*×5*^ insulator (is shown by the pale lilac arcs with blocking lines). (b) Morphology of male abdominal segments in *F6*^*1330*^ + *Pita*^*×5*^, *i5*^*1attP*^ *F6*^*1330*^ + *Pita*^*×5*^, *F6*^*1330*^ + *Pita*^*×5*^ *i6*^*1attP*^ and *i5*^*1attP*^ *F6*^*1330*^ + *Pita*^*×5*^ *i6*^*1attP*^. Schemes of the regulatory element combinations studied within the *iab-5* and *iab-6* domains are shown above the corresponding images. (c) Morphology of male abdominal segments in *Pita*^*×5*^, *Pita*^*×5*^ + *F6*^*1330*^, *i5*^*1attP*^*Pita*^*×5*^ + *F6*^*1330*^ and *Pita*^*×5*^ + *F6*^*1330*^ *i6*^*1attP*^. Designations are as in figures 2 and 3.

The results described above support the idea that initiators not only protect *iab* enhancers from PcG-mediated repression but also activate bypass modules, facilitating long-range interactions between *iab* enhancers and the *Abd-B* promoter. To further evaluate this model, we analysed how different combinations of initiators, the *F6^1330^* boundary and the *Pita^×5^* insulator influence the phenotype of male abdominal segments A5 and A6 (figure 6*a*).

First, we tested *F6^1330^ + Pita^×5^* inserted into *F6^1attP^* in combination with *i5^1attP^* and/or the *i6^1attP^* (figure 6*a**)*. In *F6^1330^ + Pita^×5^*, the *Pita^×5^* insulator separates the *iab-6* initiator from the *F6^1330^* boundary. Based on the results in Kyrchanova *et al*. [11], we would expect that *Pita^×5^* will block activation of the *Fab-6* bypass module by the *iab-6* initiator and at the same time protect from PcG-mediated repression associated with the presence of additional PRE activity inside the *iab-6* domain. As previously shown, *F6^1330^ + Pita^×5^* males (figures 4*b* and 6 and electronic supplementary material, figure S4) exhibit incomplete activation of the *iab-5* enhancers in both A5 and A6 segments, evidenced by increased trichome density on the tergites and the presence of bristles on the A6 sternite. In *F6^1330^ + Pita*^×^*^5^ i6^1attP^* males, deletion of the *iab-6* initiator does not influence the activity of the *iab-5* enhancers in the A5 segment. As expected, inactivation of the *iab-6* domain leads to a similar phenotype in both A5 and A6 segments (figure 6*a*).

In *F6^1330^ + Pita^×5^* males, pigment spots are also occasionally observed in segment A4, indicating premature activation of *Abd-B* by the *iab-5* enhancers (electronic supplementary material, figures S1 and S4). This may be explained by reduced PcG repression in *F6^1330^ + Pita^×5^*, where the PREs within *F6^1330^* and the *iab-6* domain are separated by *Pita^×5^*. Consequently, the *iab-5* initiator and enhancers are not effectively repressed in A4, leading to ectopic *Abd-B* expression. Surprisingly, deletion of either the *iab-5* initiator (*i5^attP^ F6^1330^ + Pita^×5^*) or the *iab-6* initiator (*F6^1330^ + Pita^×5^ i6^1attP^*) does not eliminate the ectopic pigmentation in A4 (electronic supplementary material, figure S4). Only the simultaneous deletion of both initiators in *i5^attP^ F6^1330^ + Pita^×5^ i6^1attP^* males leads to a complete loss of ectopic pigmentation in the A4 segment and a full transformation of segments A5 and A6 into copies of A4 (figure 6*a* and electronic supplementary material, figure S4). These results support the previous conclusion that premature activation of the *iab-6* initiator may enable the *iab-5* enhancers to stimulate *Abd-B* expression in the A4 segment.

Next, we generated transgenic *Pita^×5^+F6^1330^* flies in which the order of *F6^1330^* and *Pita^×5^* was reversed (figure 6*b*). In this arrangement, the *Pita^×5^* insulator separates the *iab-5* domain (initiator and enhancers) from the *Fab-6* boundary. In *Pita^×5^ + F6^1330^* males, the A5 segment is transformed into a copy of A4. This finding is expected since *Pita^×5^* blocks the *iab-5* initiator from activating the *Fab-6* bypass module in A5. Meanwhile, the A6 segment retains its identity and develops normally, similar to *wt* males. This result suggests that the *iab-6* initiator activates the *Fab-6* bypass module in A6 in the *Pita^×5^ + F6^1330^* replacement, and the bypass element, in turn, is able to link enhancers in the *iab-5* domain to the *Abd-B* promoter.

To confirm this model, we deleted the *i6* initiator in *Pita^×5^+F6* flies (figure 6*b*). In the resulting *Pita^×5^ + F6^1330^ i6^1attP^* males, both A5 and A6 segments were transformed into copies of A4, indicating complete inactivation of the *iab-5* enhancers. This demonstrates that the *iab-6* initiator is essential for the activity of the *iab-5* enhancers in *Pita^×5^ + F6^1330^* flies. Next, we deleted the *iab-5* initiator in the *Pita^×5^ + F6^1330^* line (*i5^1attP^ Pita^×5^ + F6^1330^*). In *i5^1attP^ Pita^×5^ + F6^1330^* males, the A6 segment retained the *wt* phenotype. Thus, despite the absence of the *iab-5* initiator, the *iab-5* enhancers are fully active in the A6 segment. This suggests that in *Pita^×5^ + F6^1330^* flies, the *Pita^×5^* insulator protects the *iab-5* enhancers from PcG-mediated repression that is linked to the *Fab-6* boundary and functions to keep the *iab-5* domain repressed. As a result, the *iab-5* enhancers are active even when the *iab-5* initiator is deleted. Taken together, these results indicate that the *iab-6* initiator controls the ability of the *iab-5* enhancers to activate *Abd-B* in the A6 segment.

## 3. Discussion

Unlike other previously studied boundaries in the BX-C complex, the *Fab-6* boundary has a unique structure: two binding sites for the architectural protein dCTCF are located within a PRE, and part of the bypass module overlaps with sequences that recruit PcG proteins. In addition, previous studies [86] showed that the *Fab-6* boundary element is a weak insulator and that an additional PRE located within the *iab-6* domain, 4 kb away, is necessary for full boundary function.

The *Fab-6* bypass module differs from the *Fab-7* and *Fab-8* bypass modules. Their bypass modules map to a DNA sequence of approximately 200 bp [80,81] that is bound by a multicomponent protein complex called the LBC, which includes the GAF and Mod(mdg4) [98,99] proteins. Like the GAF-dependent tethering elements [100], these bypass elements generate prominent contact foci in Micro-C experiments [16,17]. Mod(mdg4), which, like GAF, has an N-terminal BTB domain that forms hexamers, is presumably involved in organizing long-range interactions between regulatory elements [101,102]. The identities of other LBC components remain unknown. Importantly, ChIP-seq data do not show binding of GAF or Mod(mdg4) to *Fab-6* sequences, suggesting that a different complex mediates the activity of the *Fab-6* bypass module. Recent studies indicate that PRE-associated PcG proteins can form loops with each other and with putative enhancers, implying that PREs may function as tethering elements [21,22]. Although the *Fab-6* bypass module partially overlaps with PRE sequences, it remains functionally active even after deletion of the Pho binding sites that are responsible for PcG protein recruitment. The truncated bypass module also retains activity when part of its sequences is removed from the proximal end, provided that the dCTCF binding region is preserved. Thus, the *Fab-6* bypass module consists of several subdomains that function in cooperation with each other. Since dCTCF is also associated with the *Abd-B* promoter, it may help establish long-distance interactions between the *Fab-6* bypass element and the promoter.

**Figure 7.**
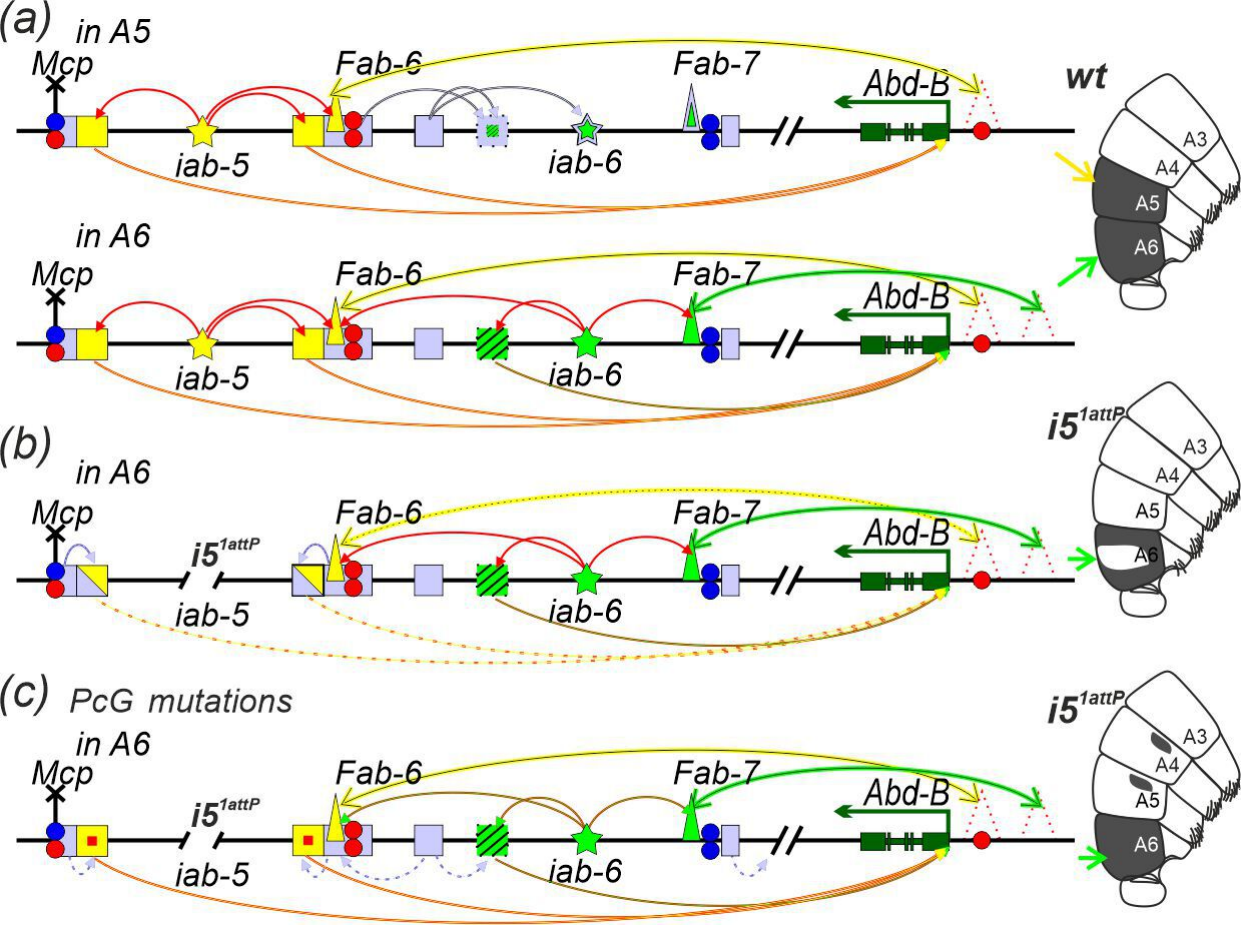
Model of the mechanism of activation of the *iab-5* and *iab-6* domains in the A5 and A6 segments. Schematic representation of Abd-B activation in (*a*) *wt* males, (*b*) *i5*^*1attP*^ males and (*c*) *i5*^*1attP*^ males crossed into a PcG mutant background. The red arrows above the diagram indicate the transmission of the active signal from the initiator to the enhancers and the bypass module within the domain. The yellow and green arrows above the diagrams indicate the interaction of the bypass module of *Fab-6* and *Fab-7* (respectively) with unmapped tethering elements (depicted as dotted triangles) in the *Abd-B* promoter region. The suppression of the *iab-6* initiator and enhancer by the *Fab-6* PRE is shown by the pale lilac arrows. The arrows below the diagrams represent the *Abd-B* activation by the *iab-5* and *iab-6* enhancers. Dotted arrows showing variable *Abd-B* activation. Squares partially shaded in pale lilac denote enhancers that are variably inactivated by PcG proteins. A small red square indicates derepression of enhancers due to PcG mutations. The inability of PRE silencers to suppress enhancers is shown by the pale lilac dotted arrows. Other designations are as in figure 2.

According to our results, the *Fab-6* boundary plays a key role in repressing the *iab-5* enhancers and the *iab-5* initiator in the A4 abdominal segment. Interestingly, our data (figure 4–6 and electronic supplementary material, figure S4) also suggest that the *Fab-6* boundary may contribute to repression of the *iab-6* initiator in the A4 and A5 segments. Thus, the *Fab-6* boundary serves three functions: it blocks crosstalk between the *iab-5* and *iab-6* domains, contributes to repression of these domains and organizes specific long-range contacts with the *Abd-B* promoter (figure 7*a*).

Overall, our results support the following model for the regulation of *iab-5* and *iab-6* domain activity. In the A5 segment, the *iab-5* initiator is activated, leading to suppression of PcG proteins bound to the *Fab-6* boundary and stimulation of the bypass module, which establishes contacts with an unidentified tethering element in the *Abd-B* promoter region (figure 7*a*). Consequently, *Abd-B* is activated by the *iab-5* enhancers, resulting in a reduction in trichome density and the male-specific pigmentation of the A5 tergite. Deletion of the *iab-5* initiator causes a partial repression of the *iab-5* enhancers by PcG proteins and prevents the *Fab-6* bypass module from targeting the *iab-5* enhancers to the *Abd-B* promoter (figure 7*b*). As a result, *Abd-B* is not expressed in A5, which is then transformed into A4. In the A6 segment, the *iab-6* initiator activates the bypass modules of the *Fab-7* and *Fab-6* boundaries, facilitating closer contact between the *iab-5* and *iab-6* enhancers and the *Abd-B* promoter, thus activating *Abd-B* (figure 7*a,b*). When the *iab-5* initiator is deleted, PcG proteins partially repress the *iab-5* enhancers, causing mosaic *Abd-B* expression in A6 (figure 7*b*). However, if PcG-mediated repression is partially relieved, the activity of the *iab-5* enhancers in A6 is restored (figure 7*c*).

Thus, the regulation of *iab* domain activity depends on the ability of initiators to activate bypass modules, which establish contacts with the promoter region and enable the *iab* enhancers to stimulate the *Abd-B* promoter. Our results demonstrate that the *Abd-B* regulatory region serves as a valuable model for studying the mechanisms of long-range contact formation and their regulation. In the future, it is crucial to identify the complexes that bind to the bypass modules in the *Fab-6* and *Fab-7* boundaries and to understand how they mediate long-distance interactions with tethering elements in the *Abd-B* promoter region. It will also be important to identify the protein complex that binds to the initiators and governs the activation of the bypass modules. This knowledge will offer experimental avenues to address the fundamental question of how long-range interactions are regulated during *Abd-B* activation—and likely more broadly in higher eukaryotes.

## 4. Material and methods

### 4.1. Generation of transgenic lines carrying different deletions and insertions

The generation of the *F6^1attP^* and *i5^1attP^* deletions was described previously [86,88]. For the generation of *F6^3attP^* and *i6^1attP^* deletions, we used CRISPR/Cas9-induced homologous recombination (electronic supplementary material, material and methods). The breakpoints of the *F6^3attP^* deletion: 3R: 1 68 83 201..16879309 according to Genome Release r6.36. The breakpoints of the *i6^1attP^* deletion: 3R: 1 68 91 553..16893019 according to Genome Release r6.36.

The strategy of the *F6^1attP^* and *F6^3attP^* replacement lines is described previously [86]. The *Fab-6* fragments were obtained by PCR amplification (electronic supplementary material, table S1). Integration of the plasmids in the *F6^1attP^* and *F6^3attP^* landing platforms was obtained by injecting the plasmid and a vector expressing the *фC31* recombinase (Addgene plasmid #26290) into *F6^1attP^* or *F6^3attP^* embryos. The successful integrations were selected on the basis of expression of the *mini-y* reporter in abdominal segments. The *mini-y, mCherry* and plasmid sequences were excised by crossing with *y^1^ w^67c23^; sna^Sco^/CyO, P{w^+mC^=Crew}DH1* (BL1092, Bloomington Drosophila Stock Centre) that expresses Cre recombinase constitutively in both germline and somatic tissue and mediates recombination between the *lox* sites. All obtained fly lines are available upon request.

### 4.2. Fly stocks and culture

Flies were grown in standard cornmeal yeast extract medium at 25°C. We used the following stocks during this study: *w^1^; TM3, ry[RK] Sb[1] Ser[1]/TM6B, Tb[1]* (BL#120 of the Bloomington Drosophila Stock Center); *In(1)ph^410^, ph-p410 w^1^* (BL#5813 of the Bloomington Drosophila Stock Center); *Pc^XT109^* [94]; KrGFP-TM3 Sb balancer (BL#5195 of the Bloomington Drosophila Stock Center); *E(z)^61^* [95].

### 4.3. Cuticle preparations

Adult flies (2–5 days) were collected in 1.5 ml microcentrifuge tubes, added with approximately 500 µl of 70% ethanol and kept for at least 20 h. Ethanol was replaced with 10% KOH and incubated at 70°C for approximately 1 h. After this, KOH was removed, washed twice with distilled water and incubated in distilled water at 70°C for approximately 45 min. The cuticles were then washed with 70% ethanol and stored in 70% ethanol. Using fine tweezers and an insulin syringe needle, the ventral part of the cuticle was separated and placed on a glass slide in a drop of glycerol. The needle was used to make a longitudinal incision along the dorsal side along the entire abdomen and cuts between the tergites. The cuticle was straightened on a glass slide by adding a drop of mineral oil and pressed with a cover glass. The preparations were photographed. The photographs presented in the work were taken using a Nikon SMZ18 stereomicroscope on a Nikon DS-Ri2 camera. Phenotypes depicted are representative of the genotypes shown. For each transgenic line, visual analysis was performed on approximately 50–100 males over several generations. In the case of a variable phenotype, the three most phenotypically different males were selected. If there were no statistically significant differences in the cuticle, the average representative cuticle was selected for display in the figures.

### 4.4. Embryo immunostaining

*Drosophila* embryos were collected 18–22 h after egg laying and dechorionated using 50% bleach. The embryos were fixed for 25 min in 4% paraformaldehyde solution in phosphate-buffered saline (PBS) with an equal volume of heptane added and then transferred to methanol for storage. The fixed embryos were stained with antibody as described earlier [103,104]. Briefly, embryos were permeabilized for 2 h with 1% Triton X-100 in PBS and then non-specific staining was blocked with a mixture of 5% bovine serum albumin and 0.1% Tween-20 in PBS. The primary antibodies were mouse monoclonal antibodies against Abd-B (1A2E9, generated by S. Celniker, deposited to the Developmental Studies Hybridoma Bank). The secondary antibodies were goat anti-mouse Alexa Fluor 594 (Thermo Fisher Scientific). At least five embryos from stage 14 of each genotype were examined, and the most representative embryos for each transgenic line were selected for presentation in the manuscript. The images were acquired on a Leica Stellaris 5 confocal microscope and processed using ImageJ software.

## Data Availability

All data needed to evaluate the conclusions in the paper are present in the paper and/or the electronic supplementary material [105]. Additional data related to this paper may be requested from the authors.
